# Calcaneal CT is a useful tool for identifying Achilles tendon disorders: a pilot study

**DOI:** 10.1186/s13018-017-0638-4

**Published:** 2017-09-25

**Authors:** Krzysztof Ficek, Jolanta Filipek, Joanna Ficek, Małgorzata Muzalewska, Filip Humpa

**Affiliations:** 1 The Jerzy Kukuczka Academy of Physical Education, Mikolowska 72A, 40-065 Katowice, Poland; 2 Galen-Orthopaedics, Jerzego 6, 43-150 Bierun, Poland; 3 Faculty of Mechanical Engineering, Silesian University of Technology, Konarskiego 18A, 44-100 Gliwice, Poland; 4 Galen Rehabilitation, Jerzego 6, 43-150 Bierun, Poland

**Keywords:** Calcaneus, Achilles tendon, Computed tomography

## Abstract

**Background:**

In various Achilles tendon disorders, little attention is paid to the bone environment at the tendon insertion sites. The aim of the present study was to assess the calcaneal bone structure in Achilles tendon disorders using computed tomography (CT).

**Methods:**

This study included 31 male patients diagnosed with various Achilles disorders, including episodes of tendon rupture (TR), conservatively treated tendinopathy (TP), and critical-stage Achilles TP treated with endoscopic surgery (TS). CT scans of both feet were conducted to assess the calcaneal bone structure in the TP and TS groups, which comprised 23 patients. Bone measurements were calculated, including the bone volume-to-total volume ratio (BV/TV), cross-sectional area (CSA), product moment of area (Ipm), and polar section modulus (Zpol).

**Results:**

The results demonstrated increased BV/TV, CSA, Ipm, and Zpol values in patients who underwent tendinoscopy and in patients with insertional TP.

**Conclusions:**

CT images are useful for evaluating calcaneal trabecular structural alterations in patients with Achilles pathology and correlate with the TP type.

## Background

Degenerative tendinopathy, i.e., tendinosis [1], refers to intrinsic deformations in the structure of Achilles tendon fibers without self-reported patient symptoms or subjective pain. This condition does not involve inflammation or clinical manifestations but may develop into active tendinopathy (TP) with variable clinical symptoms, which are usually localized in the narrowest cross-sectional segments (midportion Achilles TP, 2–7 cm above the calcaneal insertion) [2]. In contrast, 20% of Achilles disorders involve insertional tendinopathy [2], which is defined as a degeneration of the fibers of the Achilles tendon at its insertion into the calcaneus. Undoubtedly, the deterioration of tendinous structures suggests an approach for diagnostic development. Among the well-known imaging techniques, radiography and MRI appear sufficient for detecting soft tissue and bone changes, such as heel spurs or Haglund’s deformity. The use of computed tomography (CT) is uncommon in cases of both tendinopathy and Achilles tendon rupture (TR). However, CT provides an opportunity to precisely evaluate the quality of the calcaneal bone and its simultaneous anatomical relationship with the Achilles tendon.

Among various Achilles tendon pathologies and perspectives regarding its structural and functional anatomy, the calcaneal bone environment and tendon insertion sites have received scant attention. Generally, although its fibrous structure has been considered [3–6], little discussion has addressed the specific attachment of the Achilles tendon [7]. The tendon-bone insertion is characterized as a complex composite junction that allows stress transfer between mechanically dissimilar materials. Bone is a stiff, brittle material; in comparison, tendon has a material stiffness of approximately 20 GPa [8]. In contrast, tendon is tough and extensible, while bone has a material stiffness of approximately 200 MPa in tension [9]. The attachment of two dissimilar materials results in stress singularities at their interface and a subsequent increased risk of failure [10]. The mechanical response at the tendon-bone interface in the transverse direction enables the directional variability of the force applied to the bone [5]. The concentration of trabeculae and an increase in bone density (radiologically described as bone sclerosis) within the calcaneus and particularly around the tendon-to-bone attachment may affect the Achilles tendon’s interaction with fibrous tissue. Therefore, in probing the causes of emerging tendinopathy, the interaction between the diverse environments of tendon and bone merits investigation that considers appropriate transmission loads for the tendon and stress absorption for the bone.

The structural behavior of the bone is affected by both bone geometry (which reflects different geometric arrangements of the bone tissue) and material properties. There are several aspects of bone cross-sectional geometry, such as the cross-sectional area and the moment of inertia, that are useful for predicting structural properties [11]. The cross-sectional area (CSA), product moment of area (Ipm), and polar section modulus (Zpol) are often calculated to measure bone strength [12–15]. The CSA measurement of the calcaneus provides information about the distribution of bone minerals [16]. In bending, the strength and stiffness of bones are determined by Ipm [17], and Zpol is a measure of torsional strength [18].

We hypothesized that the inner architecture of the calcaneus is related to Achilles tendon pathology. The main goal of this study was to determine the structural parameters of the calcaneus and their association with disorders of the Achilles tendon.

## Methods

### Diagnostics

Thirty-one male patients (average age, 46 years; range, 21 to 73 years) undergoing treatment for various Achilles tendon disorders at the Galen-Orthopaedics Clinic, Bierun, Poland, were initially considered for the study. The patients’ diagnoses included episodes of TR, TP treated with conservative management, and critical-stage Achilles TP treated with endoscopic surgery (TS). The epidemiology is shown in Table 1. All the TR patients had a spontaneous rupture that occurred during physical activity. The TP and TS patients experienced episodes of pain for at least 3 months before notifying the physician. In eight patients, TP occurred bilaterally; seven patients did not report any symptoms in the other leg (Table 1). The orthopedic examination included routine palpation of the tendon, a range of motion assessment, and ultrasonography. During the medical interview, patients self-reported their activity levels. Patients with ruptures of the Achilles tendon were excluded from the CT analysis due to the various times after percutaneous surgery using the Ma-Griffith technique (up to 2 years). During this post-surgical period, the calcaneus can undergo unpredictable conversion as a result of unilateral loading. Therefore, 23 patients (Table 2; average age, 45 years; range, 21 to 67 years) were included for CT examination and further evaluation.

**Table 1:** The different combinations of bilateral calcanei in 31 patients

LL	Contralateral LL	Patients (*n*)
TP	TP	8
TP	NS	7
TP	TS	1
TP	TR	2
TR	NS	6
TS	NS	6
TS	TS	1
Total		31

*LL* lower limb without distinction between left and right limbs, *NS* no symptoms, *n* number of patients

**Table 2:** The different combinations of bilateral calcanei in 23 patients included for CT examination

LL	Contralateral LL	Patients (*n*)	No. of calcanei with TP	No. of calcanei with TS	No. of calcanei with NS
TP	TP	8	16	–	–
TP	NS	7	7	–	7
TP	TS	1	1	1	–
TS	NS	6	–	6	6
TS	TS	1	–	2	–
Total		23	24	9	13

*LL* lower limb without distinction between left and right limbs, *NS* no symptoms, *n* number of patients

The control group (CG) consisted of nine male individuals (average age, 39.8 years; range, 31 to 53 years) without any history of Achilles tendinopathy. The characteristics of the subjects included in this study are presented in Table 3.

**Table 3:** Subject characteristics

	CG	TP	TS	MP	INS
Age (years)	39.8 ± 7.1	48.9 ± 9.9	35.1 ± 9.6	51.1 ± 11.0	40.1 ± 11.3
Height (cm)	181 ± 7	179 ± 6	181 ± 6	178 ± 8	181 ± 5
Weight (kg)	86 ± 11	86 ± 14	84 ± 7	83 ± 9	86 ± 13
BMI	26.2 ± 2.0	26.8 ± 3.8	25.6 ± 1.9	26.4 ± 3.5	26.4 ± 3.3

CT scans (GE BrightSpeed Elite, GE Healthcare, US) of both feet were performed to assess the calcaneal bone structure. A CT reconstruction protocol was established to provide comparable images. The slice thickness of the sagittal cross sections was 1 mm; pixel spacing, 0.33/0.33; and convolution kernel: bone, 100 kV. CT scans of the CG were also performed to assess bone structure among individuals with no Achilles tendon symptoms.

The calcanei of the 23 patients were divided in the following two ways: by treatment (tendinopathy treated with conservative management (TP) [*n* = 24]; TP treated with endoscopic surgery (TS) [*n* = 9]) (Table 2), or by anatomical location (midportion (MP) [*n* = 11]; insertional (INS) [*n* = 22]). The division by anatomical location was performed by one orthopedic specialist based on the localization of Achilles tendon pain and swelling (midportion TP, 2–7 cm above the calcaneal insertion, insertional TP < 2 cm). Calcanei from the NS group (Table 2) were not included in the inner bone structure analysis.

Approval for this study was granted by the ethics committee under agreement number KB/13/2007.

### Image analysis

**Fig. 1:**
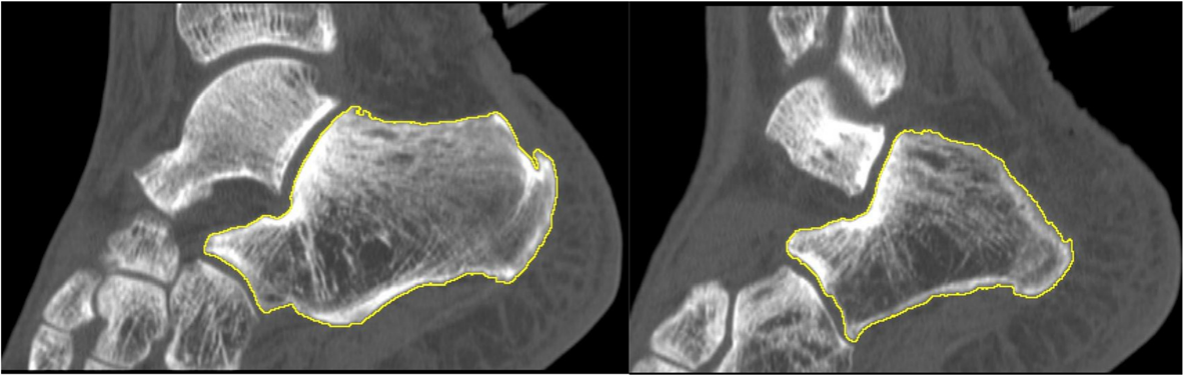
Calcaneal region of interest (ROI) constructed on various cross sections

Based on the CT images, bone measurements were performed using ImageJ [19], and the bone structures were analyzed using the BoneJ plugin [20]. Two observers (biomedical engineering specialists) independently applied the segmentation procedure to sagittal cross sections of bone tissue from a stack of images to obtain interobserver reliability. The following threshold was then averaged, and the mean value was used in the following measurements. Independently, calcaneal regions of interest (ROIs) were manually constructed for each slice by one observer (Fig. 1). The ratio of the bone volume to the total volume (BV/TV), which indicates the percentage of mineralized bone located within the volume of interest, was calculated based on binarized images. New stacks, with the images centered and rotated so the principal axes were parallel to the *x*-, *y*-, and *z*-axes of the image stack [20], were automatically generated (via the BoneJ plugin) for each calcaneus prior to performing the slice geometry measurements. The CSA, Ipm, and Zpol were calculated to measure bone strength.

**Fig. 2:**
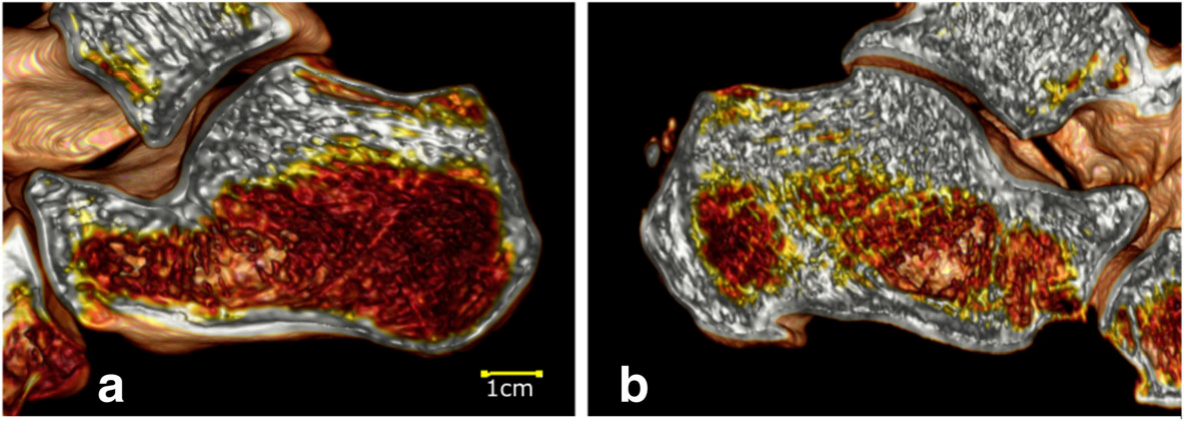
The figure shows 3D visualizations of calcanei with bone tissue distributions: **a** calcaneus from the CG and **b** calcaneus from the TS group. White color represents bone tissue of high density; red color represents bone tissue of low density

An open-source platform, OsiriX MD (v. 7.5, Osiris Foundation, Geneva, Switzerland), was used for image processing and for the 3D analysis of inner bone structures (Fig. 2). The OsiriX volume-rendering algorithm was used to produce a 3D reconstruction of the calcaneus. High contrast was automatically applied to select bone density and only show dense bone structures. Additionally, the level of detail for the volume rendering was set, and high-quality images were obtained.

### Statistical analysis

Statistical analysis was conducted in consideration of two aspects: treatment (analysis A) and anatomical location (analysis B). The following tests were performed for each grouping. The Shapiro-Wilk test and Levene’s test were used to assess data normality and variance homogeneity, respectively. Despite unequal sample sizes, the results indicated that insufficient evidence existed to reject either the hypothesis of normality or the hypothesis of variance homogeneity. Because the distribution of Zpol was skewed, the values were log-transformed. One-way ANOVA was used for overall group comparisons, followed by the Tukey HSD post hoc test for group-wise comparisons; *p* < 0.05 was considered significant.

A sensitivity analysis of one foot per patient was performed to inspect the influence of the correlation between measurements of the same patient.

## Results

**Fig. 3:**
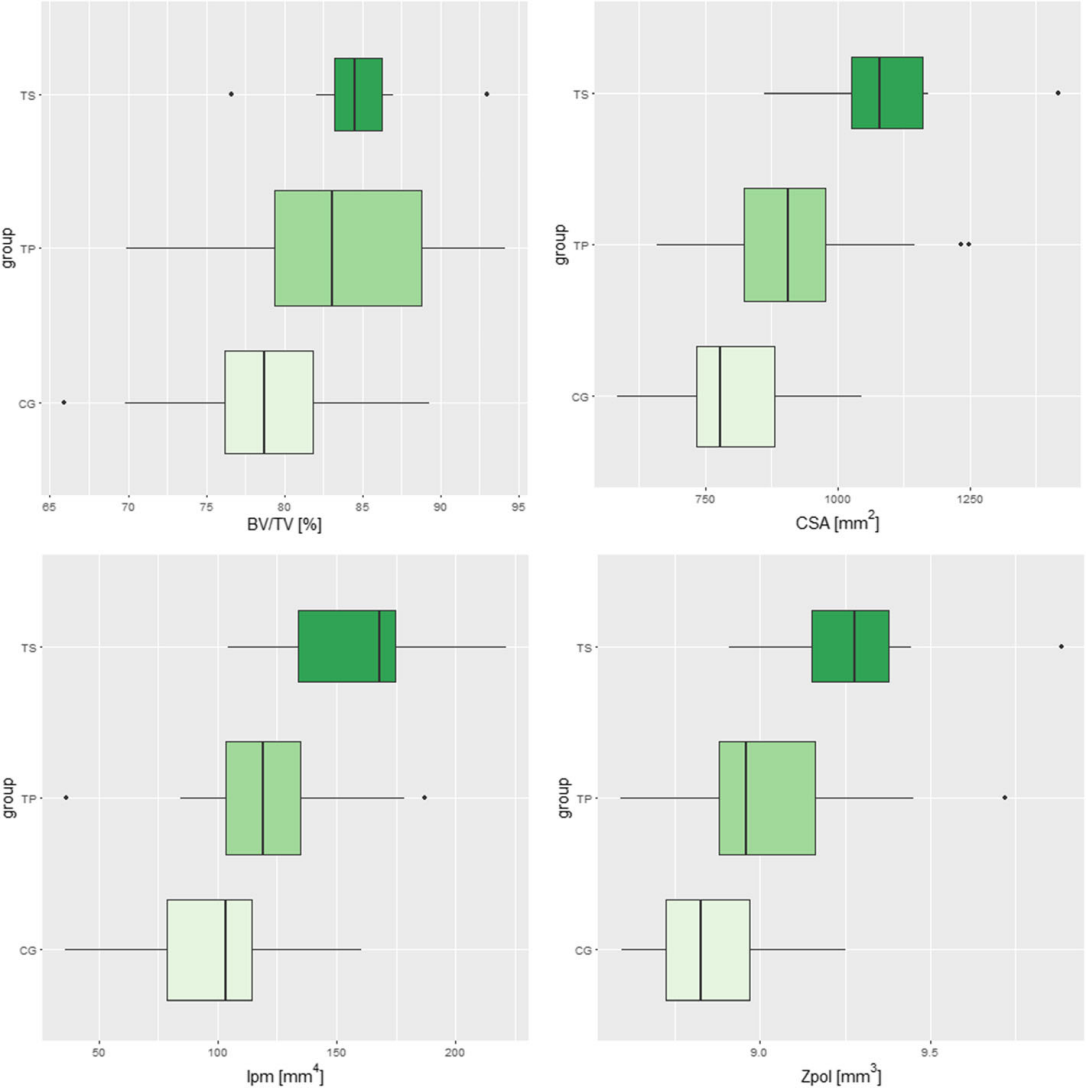
Boxplots showing the calcaneal bone structure measurement results from analysis A. Boxplot legend: the top and the bottom of the boxes represent the 1st and 3rd quartiles, respectively. The whiskers correspond to the maximal and minimal value within the 1.5 × inter-quartile range (IQR). Outliers are plotted as points

Agreement between two observers (interobserver agreement) regarding the thresholding of calcaneal bone was established for 79.66% of calcanei examined. The comparison of the cross-sectional geometric properties of the calcaneus between the tested groups in analysis A is shown in Fig. 3 and Table 4. The highest average BV/TV was observed in the TS group. Compared with the CG and the TP group, the TS group demonstrated a higher CSA. Additionally, the following significant differences in CSA were found: CG-TP (*p* = 0.03233), CG-TS (*p* = 0.00004), and TP-TS (*p* = 0.01286). The analysis also revealed significant differences in Ipm (CG-TS [*p* = 0.00011], TP-TS [*p* = 0.01965]) and Zpol (CG-TS [*p* = 0.00063], TP-TS [*p* = 0.04024]).

**Table 4:** Results from analysis A

	Analysis A			
	ANOVA	CG-TP	CG-TS	TP-TS
BV/TV	0.03465	0.06694	0.07168	0.86550
CSA	0.00006	0.03233	0.00004	0.01286
Ipm	0.00017	0.05117	0.00011	0.01965
Zpol	0.00096	0.11017	0.00063	0.04024

**Fig. 4:**
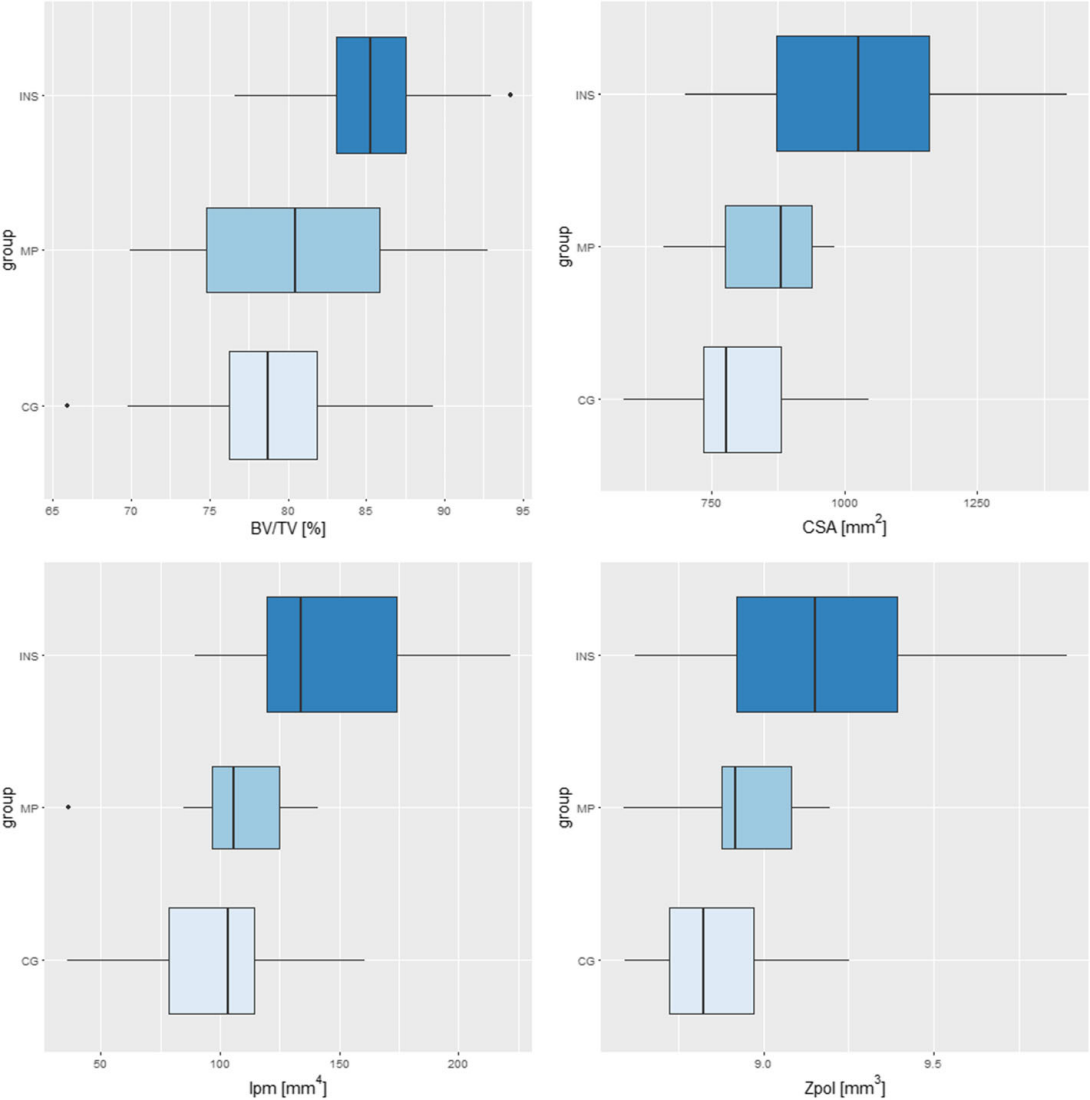
Boxplots showing the calcaneal bone structure measurement results from analysis B. Boxplot legend: the top and the bottom of the boxes represent the 1st and 3rd quartiles, respectively. The whiskers correspond to the maximal and minimal value within the 1.5 × inter-quartile range (IQR). Outliers are plotted as points

The comparison of the bone structure parameters between the tested groups in analysis B is provided in Fig. 4 and Table 5. The highest values of BV/TV, CSA, Ipm, and Zpol were observed in the INS group, and the lowest values were found in the CG. The following significant differences were found between groups: CG-INS (*p* = 0.00282) for BV/TV; CG-INS (*p* = 0.00004) and MP-INS (*p* = 0.00840) for CSA; CG-INS (*p* = 0.00008) and MP-INS (*p* = 0.00788) for Ipm; and CG-INS (*p* = 0.00089) and MP-INS (*p* = 0.03799) for Zpol (Table 5).

**Table 5:** Results from analysis B

	Analysis B			
	ANOVA	CG-MP	CG-INS	MP-INS
BV/TV	0.00265	0.81037	0.00282	0.05327
CSA	0.00004	0.56185	0.00004	0.00840
Ipm	0.00007	0.68381	0.00008	0.00788
Zpol	0.00091	0.70459	0.00089	0.03799

The 3D CT reconstructions derived from Digital Imaging and Communications in Medicine (DICOM) images clearly visualized the interior architecture, which was difficult to analyze based on standard 2D images (Fig. 1). Among individuals, such 3D models displayed various cortical and trabecular configurations, particularly in sites adjacent to the Achilles tendon insertion site (Fig. 2). Among the calcanei from the INS group (*n* = 22), bone sclerosis was not visualized in only three cases but was observed in six cases in the TS group (*n* = 9); however, such 3D examination revealed various sclerotic patterns in relation to location and range.

## Discussion

In this study, we proposed the observation of bone alterations in patients with an Achilles pathology using CT to estimate the bone structure and identify associations between the inner calcaneal architecture and the occurrence of Achilles disorders. CT allows a close examination of the bone distribution within the calcaneus and the possibility of identifying bone sclerosis at the Achilles attachment site. Structurally, bone strength and stiffness depend on the CSA for tension and compression and on the distribution of bone mass about the neutral axis, which is known as the Ipm or area moment of inertia [17]. Zpol, which is a measure of torsional strength, may also be determined to estimate overall average bending strength [21]. The results of the CT analysis demonstrated that calcanei from the TS and INS groups were characterized by higher values in the parameters (CSA, Ipm, Zpol) that describe calcaneus strength and stiffness; however, cross-sectional geometrical properties are usually used to test the mechanical adaptations of long bones and to reflect the resistance of bone to axial compression and tension [22]. Nevertheless, the calcaneus is a structure that is subjected to complex loading and reflects the combined action of the forces applied. The combination of compressive forces, shear stress, torsion, and tensile stress, which is imposed at the insertion of the Achilles tendon and plantar fascia [23], act on the calcaneus during standing. This study verifies the utility of cross-sectional parameters (CSA, Ipm, Zpol) in calcaneus measurements because calcaneal bone is also subjected to compression and tension. Typically, a detailed assessment of trabecular bone in the calcaneus, obtained by calculating trabecular thickness, trabecular number, connectivity, etc., may be obtained via a micro-tomographic evaluation; however, such estimation of bone structure is infeasible in vivo. Between CG and TS, in which 67% cases had radiologically indicated bone sclerosis, significant differences (*p* < 0.05) were observed in CSA, Ipm, and Zpol. Concerning the division of Achilles tendinopathy by localization: midportion and insertional, we showed that insertional tendinopathy is associated with changes in calcaneal bone inner structure, based on increased values for parameters such as CSA, Ipm, and Zpol in the INS group. Statistically significant results were observed in the calculated parameters between the CG and INS groups. The lack of significant differences between the CG and MP suggest that the etiology of midportion Achilles tendinopathy is poorly associated with calcaneal bone tissue. Therefore, our hypothesis regarding the association of the inner architecture of the calcaneus with Achilles tendon pathology was partially confirmed. To account for the correlation between measurements of the same patient’s feet, a sensitivity analysis was performed in which one foot per patient was randomly chosen for the analysis. The results are in line with those presented here for the whole dataset. Although the order of the *p* values slightly increased as they strongly depend on the sample size, the significance of the results remained unchanged. Moreover, the authors emphasize that this is a pilot study and hence, significance should not be the only factor considered when assessing the results, which should be accompanied by visual inspection (see Figs. 3 and 4).

Based on clinical history, patients involved in various sports (e.g., soccer, triathlon, tennis) who also met the World Health Organization’s (WHO) recommended physical activity levels for adults [24] were identified. Sixteen of the 23 patients were involved in various sports activities (e.g., soccer, triathlon, tennis, running). To compare the influence of sport loading on calcaneal bone inner structure, we decided to include individuals without a history of Achilles tendon pathology (i.e., the CG, *n* = 9) who also engaged in high-intensity exercises and met the WHO’s recommended levels of physical activity. In two cases in the CG, the evaluation of the inner calcaneal bone structure indicated increased density. Such a finding raises questions about using CT to predict Achilles tendon disorders, particularly insertional pathologies, which may be caused by insufficient absorption of the trabecular bone tissue within the calcaneus. Therefore, to explore and challenge the traditional hypothesis regarding the etiology of Achilles TP, further research including larger groups of patients should be performed. In light of the above considerations, all the collected samples that met the basic requirements were included in this pilot study. To adjust for the similarity between the measurements of both feet of the same patient, the sample size was reduced to one randomly chosen foot per person for the sensitivity analysis. This analysis confirmed the results presented above.

The role of the hard-soft tissue interface is commonly neglected when considering the factors that may affect the calcaneal tendon. Hems and Tillmann [25] highlighted that bone and tendon have similar tensile strengths but different elastic moduli, the latter of which is nearly 10 times higher in bone. Additionally, Suresh [18] emphasized that difficulties arise from joining a stiff material (bone in the context of the enthesis) to a softer one (tendon).

Biomechanical changes in the tendinopathic tendon with the assessment of ultimate tensile strength appear to be well established in the literature [26, 27]. The vast majority of research has focused on Achilles tendon transformations resulting from soft tissue changes. The Achilles tendon becomes stiffer with chronic loading and non-uniform stress distribution within the tendon, which leads to a microtrauma effect between fibrils [28, 29]. Therefore, the conversion from deteriorated tendon fibrils to mineralized fibro-cartilage may cause outer bone deformations, such as bone spurs or Haglund’s deformity. Because of the association between the Achilles enthesis and Sharpey’s fibers, the postero-superior prominence of the calcaneus must also be investigated [30]. Because bone is stiffer than a tendon-muscle system, the dispersion of stress from bone to tendon must be noticeable, considering the two types of tendon insertion into bone described in the literature, i.e., indirect (periosteal-diaphyseal) and direct (chondral-apophyseal) [31].

In addition to external heel alterations, the role of the inner structure of bones in the remodeling process should be considered. Remodeling may be induced by transmitted and improperly absorbed loads from tendons. Tensile stress fractures may be generated in the calcaneus adjacent to the Achilles tendon insertion [32], and localized bone sclerosis may be caused by microinjuries that compress the bone [33]. Such an increase in bone density, assuming further interaction between the calcaneus and tendon, might diminish the stress absorption capability of the bone and consequently induce further degeneration of the tendon fiber structure. More than a century ago, Wolff [34] postulated that bone tissue responds to its environment, causing trabecular architecture remodeling. To date, few studies have verified the Achilles tendon’s influence on cancellous tissue. Biewener et al. [35] assessed the Achilles tendon’s impact in an animal model (potoroo) using fluorescence microscope to analyze the histological organization of trabeculae. The results tentatively suggested that variations in Achilles tendon anatomy might affect the trabecular structure of the calcaneus. Additionally, Kuo et al. [36] attempted to investigate the correlation between bone parameters and Achilles morphology in primates using micro-CT. To the authors’ best knowledge, no studies to date have verified these assumptions in human subjects while measuring bone inner structure using such parameters as BV/TV, CSA, Ipm, and Zpol in calcaneus. Different parameters, such trabecular thickness (Tb.Th), trabecular spacing (Tb.Sp), and trabecular number (Tb.N), based on high-resolution images, were measured, making direct comparisons between our results and those of previous studies infeasible.

This study has several strengths and a few limitations. One strength is the novelty of proposing a new factor influencing Achilles tendon disorders. To the best of our knowledge, this is the first study to highlight the possible impact of bone in the progression of degenerative changes in Achilles tendinopathy given that bone is the structure that absorbs the transferred stresses of the working tendon. The decreased bone tissue absorption properties resulting from the increased density of the trabecular structure may lead the remaining forces to overload the tendon fibers. Despite the small number of patients in our study, we found significant differences between the control and tendinopathic Achilles tendons. The presence of localized bone sclerosis was also indicative of the severity of the disease—increased bone density was observed in patients after tendinoscopy. Additionally, the color 3D observation of the inner architecture of the calcaneus showed the distribution of trabeculae, emphasizing bone sclerosis, which is difficult to observe on 2D images. Furthermore, the analysis of the demographic characteristics of the participant groups showed no significant differences in BMI between the cohorts (CG, TP, TS (*p* = 0.9191) and CG, MP and INS (*p* = 0.9174)), considering that BMI may correlate directly and strongly with primary outcomes.

This study has some limitations. The study was not designed to determine Achilles tendinopathy causation but to establish an association between the incidence of Achilles pathology and bone properties and to indicate a new factor that might be considered in the etiology. The authors did not exclude the concept of primary calcaneal bone alteration caused by a functioning tendon. Therefore, a mutual influence between the calcaneus and tendon is likely. The results are based only on males. Although different studies report that the incidence of tendinopathy is similar in both genders [37], we observe episodes of Achilles tendon pain more frequently in males. Additionally, females have different bone metabolism at various ages than males [38]; thus, to eliminate potential confounding effects, the study was limited to males. The level of physical activity according to the WHO criteria was not scored; only a subjective evaluation was performed. A correlation between the reported activity level and the occurrence of bone sclerosis was perceived by the study radiologist based on visual observations but was not described quantitatively.

## Conclusions

Our goal was to propose a new concept in the consideration of Achilles tendon pathologies by analyzing the inner bone structure as a factor contributing to stress dissipation. The results of this study enable the consideration of an etiological model of tendinopathy involving deeper parts of the bone.

## Abbreviations

BMI: Body mass index
BV/TV: Bone volume-to-total volume
CG: Control group
CSA: Cross-sectional area
CT: Computed tomography
INS: Insertional Achilles tendinopathy
Ipm: Product moment of area
MP: Midportion Achilles tendinopathy
MRI: Magnetic resonance imaging
ROI: Region of interest
TP: Conservatively treated tendinopathy
TS: Critical-stage Achilles TP treated with endoscopic surgery
WHO: World Health Organization
Zpol: Polar section modulus
